# Estimating Central Pulse Pressure From Blood Flow by Identifying the Main Physical Determinants of Pulse Pressure Amplification

**DOI:** 10.3389/fphys.2021.608098

**Published:** 2021-02-23

**Authors:** Joaquín Flores Gerónimo, Eugenia Corvera Poiré, Philip Chowienczyk, Jordi Alastruey

**Affiliations:** ^1^Department of Biomedical Engineering, School of Biomedical Engineering and Imaging Sciences, King's College London, London, United Kingdom; ^2^Departamento de Física y Química Teórica, Facultad de Química, Universidad Nacional Autónoma de México, Ciudad Universitaria, Mexico City, Mexico; ^3^Universitat de Barcelona Institute of Complex Systems (UBICS), Universitat de Barcelona, Barcelona, Spain; ^4^Department of Clinical Pharmacology, British Heart Foundation Centre, St Thomas' Hospital, King's College London, London, United Kingdom; ^5^World-Class Research Center, Digital Biodesign and Personalized Healthcare, Sechenov University, Moscow, Russia

**Keywords:** pulse pressure amplification, 1-D model, peripheral pressure, central pressure, analytical solutions, hemodynamics

## Abstract

Several studies suggest that central (aortic) blood pressure (cBP) is a better marker of cardiovascular disease risk than peripheral blood pressure (pBP). The morphology of the pBP wave, usually assessed non-invasively in the arm, differs significantly from the cBP wave, whose direct measurement is highly invasive. In particular, pulse pressure, PP (the amplitude of the pressure wave), increases from central to peripheral arteries, leading to the so-called pulse pressure amplification (ΔPP). The main purpose of this study was to develop a methodology for estimating central PP (cPP) from non-invasive measurements of aortic flow and peripheral PP. Our novel approach is based on a comprehensive understanding of the main cardiovascular properties that determine ΔPP along the aortic-brachial arterial path, namely brachial flow wave morphology in late systole, and vessel radius and distance along this arterial path. This understanding was achieved by using a blood flow model which allows for workable analytical solutions in the frequency domain that can be decoupled and simplified for each arterial segment. Results show the ability of our methodology to (i) capture changes in cPP and ΔPP produced by variations in cardiovascular properties and (ii) estimate cPP with mean differences smaller than 3.3 ± 2.8 mmHg on *in silico* data for different age groups (25–75 years old) and 5.1 ± 6.9 mmHg on *in vivo* data for normotensive and hypertensive subjects. Our approach could improve cardiovascular function assessment in clinical cohorts for which aortic flow wave data is available.

## 1. Introduction

Peripheral systolic blood pressure is the most commonly used measure of circulatory function and cardiovascular risk. However, a significant predictive benefit has been observed when measuring central (aortic) blood pressure (cBP) (Agabiti-Rosei and Muiesan, 2015; Williams et al., 2018) and it has been suggested that cBP should be a better indicator of risk (Agabiti-Rosei et al., 2007; Avolio et al., 2009; Sharman et al., 2013; McEniery et al., 2014; Agabiti-Rosei and Muiesan, 2015; Williams et al., 2017) since it is more representative of the load exerted on major organs (Herbert et al., 2014; Agabiti-Rosei and Muiesan, 2015; Williams et al., 2017). For instance, elevation of cBP induces coronary arteriosclerosis which in turn can lead to adverse events such as stenosis and myocardial infarction (Agabiti-Rosei et al., 2007) and causes chronic kidney disease that can advance to end-stage renal disease (Safar et al., 2002; Ohno et al., 2016). Additionally, it has been suggested that cBP assessment can improve therapeutic decisions since anti-hypertensive drugs have different effects on peripheral and central pressure values (Sharman et al., 2013; McEniery et al., 2014; Agabiti-Rosei and Muiesan, 2015).

**Figure 1:**
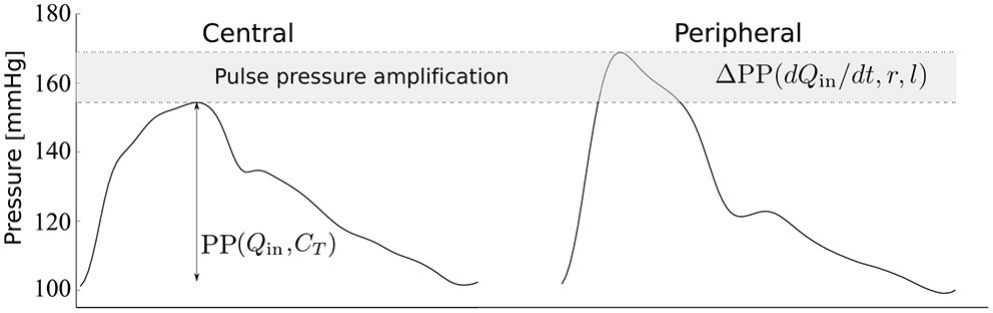
Central and peripheral pressure waveforms. Central pulse pressure, cPP, is mainly determined by the blood flow ejected by the ventricle into the aorta, *Q*_in_, and the total arterial compliance, *C*_T_ (Vennin et al., 2017). Peripheral PP amplification, ΔPP, is mainly determined by the rate of change of *Q*_in_ with time, *t*, in late systole and the radius, *r*, and length, *l*, of the brachial artery, as shown in this study.

The morphology of the peripheral blood pressure (pBP) wave, usually assessed non-invasively in the arm, differs significantly from the morphology of the cBP wave, whose direct measurement is highly invasive. In particular, pulse pressure (PP)—the difference between systolic and diastolic blood pressures—often increases from central to peripheral arteries leading to the so-called pulse pressure amplification (ΔPP) (Figure 1). The cBP wave can be estimated using population-based generalized transfer functions (GTFs) from a calibrated pBP wave; however the debate continues on the suitability of this approach for all patients and conditions (Avolio et al., 2009; Shih et al., 2011). Several studies have proposed an adaptive transfer function technique which personalizes some of the parameters of a single-tube transmission line model coupled to an impedance boundary condition that reflects incoming pulse waves (Swamy et al., 2009; Hahn et al., 2012; Gao et al., 2016; Natarajan et al., 2017). Such an approach can improve the accuracy of the estimated cBP waveform compared to the GTF approach. In addition, the pressure wave measured in the common carotid artery has been used as a surrogate for the cBP wave; however reproducible pressure waves are more difficult to obtain at the carotid artery than at the radial artery due to anatomical reasons (Avolio et al., 2009).

Understanding the effect of cardiovascular parameters on ΔPP could improve cBP assessment from non-invasive pBP measurements; specially central PP (cPP) assessment which has been shown to be of greater predictive value for cardiovascular outcomes than brachial PP (Safar et al., 2002; Williams et al., 2006). Large ΔPP values have been associated with male sex (Segers et al., 2009; Herbert et al., 2014), higher heart rate (Wilkinson et al., 2002), height (Asmar et al., 1997; Camacho et al., 2004), mass index (Pichler et al., 2016), pulse transit time (Gao et al., 2016; Natarajan et al., 2017), and wave reflection coefficient (Gao et al., 2016), and lower age (Wilkinson et al., 2001; Herbert et al., 2014) and pulse wave velocity (Hashimoto and Ito, 2010; Pierce et al., 2012), and is significantly influenced by cardiovascular risk factors, such as hypertension and obesity (Herbert et al., 2014). Experimental and computational models have been used to study the effect on ΔPP of cardiovascular properties (Karamanoglu et al., 1995; Figueroa and Humphrey, 2014; Mynard and Smolich, 2015; Gaddum et al., 2017) and age (Charlton et al., 2019), showing that ΔPP raises with increasing ventricular inotropy (contractile state of the ventricle), tapering, peripheral load and vessel length; and decreasing wall thickness and age. However, there are currently no methods based on the physics of blood flow in the systemic arterial tree that enable explicit analytical identification of the main cardiovascular determinants of ΔPP—and hence estimation of cPP from peripheral PP (pPP)—from data that can be acquired non-invasively for a specific subject.

The main purpose of this study was to develop a methodology for estimating cPP from non-invasive measurements of blood flow (aortic or brachial) and pPP. Our novel approach is based on a comprehensive understanding—using the physics of blood flow—of the main cardiovascular properties that determine ΔPP along the aortic-brachial arterial path. The methodology presented in this study was assessed using *in silico* data generated by blood flow modeling and *in vivo* data measured in normotensive and hypertensive subjects. These datasets included reference cPP values.

## 2. Materials and Methods

### 2.1. *In vivo* Data

We used previously available measurements of cBP and pBP waveforms in a group of normotensive volunteers (*n* = 26) and hypertensive subjects (*n* = 57) (Fok et al., 2014a; Li et al., 2017). Subjects were recruited from those who were evaluated for hypertension at Guy's and St Thomas' Hypertension Clinic. Although they were referred for evaluation of hypertension, blood pressure settled in some subjects and the sample included some who were normotensive (Li et al., 2017). Subjects with significant valvular disease, impaired left ventricular systolic function (ejection fraction <45%), and arrhythmia were excluded. 60% of the hypertensive subjects were on treatment with anti-hypertensive medications. Characteristics of both groups are given in Table 1. Radial and carotid pressure waveforms were obtained by applanation tonometry performed by an experienced operator using the SphygmoCor system (AtCor, Australia). Ensemble-averaged carotid pressure was used as surrogate for ascending aortic pressure (Chen et al., 1996). Approximately 10 cardiac cycles were obtained and ensemble averaged. Brachial blood pressure was measured in triplicate by a validated oscillometric method (Omron 705CP, Omron Health Care, Japan) and used to calibrate radial waveforms and, thus, to obtain a mean arterial pressure (MAP) through integration of the radial waveform. Carotid waveforms were calibrated from MAP and diastolic brachial blood pressures on the assumption of equality of these pressures at central and peripheral sites (Pauca et al., 1992). Ultrasound imaging was performed by an experienced operator using the Vivid-7 ultrasound platform (General Electric Healthcare, United Kingdom). Velocity above the aortic valve was recorded using pulsed wave Doppler obtained from an apical 5-chamber view. All ultrasound measurements were averaged over at least 3 cardiac cycles. Cross-sectional area of the aortic valve (obtained in the parasternal long-axis view) was used to estimate the aortic radius. These data were acquired in a previous study approved by the London Westminster Research Ethics Committee, for which written informed consent was obtained (Li et al., 2017).

**Table 1:** Characteristics of the datasets: normotensive (second column) and hypertensive (third column) *in vivo* subjects and *in silico* subjects (fourth column).

**Characteristic**	**Normotensives (*n* = 26)**	**Hypertensives (*n* = 57)**	***In silico* (*n* = 4374)**
Age (years)	44 ± 14	40 ± 13	50 ± 17
Sex (male %)	58	65	100
Height (m)	1.72 ± 0.03	1.72 ± 0.09	1.76 ± 0.00
Weight (kg)	79.2 ± 7.7	79.4 ± 13.4	-
Heart rate (bpm)	62 ± 8	62 ± 10	76 ± 9
*r*_Ao_ (cm)	0.96 ± 0.12	0.98 ± 0.11	1.97 ± 0.19
*r*_Bra_ (cm)	0.20 ± 0.03	0.20 ± 0.03	0.45 ± 0.05
SV (mL)	55.4 ± 15.8	52.4 ± 13.9	60.4 ± 12.4
(Δ*Q*_in_/Δ*t*)_Ao_ (mL/s^2^)	1550 ± 464	1580 ± 405	1966 ± 270
*dQ*_in_/*dt*|_min,Bra_ (mL/s^2^)	96 ± 35	98 ± 31	434 ± 83
cPP (mmHg)	39.7 ± 12.3	45.6 ± 13.5	35.0 ± 15.3
pPP (mmHg)	51.0 ± 12.0	54.1 ± 14.7	50.2 ± 13.6
ΔPP (mmHg)	11.2 ± 6.4	11.5 ± 7.5	15.5 ± 5.2
R_*T*_ (mmHg s/mL)	1.96 ± 0.55	2.20 ± 0.70	1.27 ± 0.31
C_*T*_ (mL/mmHg)	1.15 ± 0.48	1.04 ± 0.40	0.85 ± 0.30

*Values are percentage or mean ± SD. r_Ao_, aortic radius; r_Bra_, brachial radius; SV, stroke volume; (ΔQ_in_/Δt)_Ao_, approximate maximum temporal rate of decrease in late-systolic flow at the ascending aorta; dQ_in_/dt|_min,Bra_, maximum temporal rate of decrease in late-systolic flow at the brachial artery; cPP, central pulse pressure; pPP, peripheral pulse pressure; ΔPP, pulse pressure amplification; R_T_, total vascular resistance; C_T_, total vascular compliance. r_Bra_ and dQ_in_/dt|_min,Bra_ for the in vivo datasets were estimated from the corresponding data in the aorta using Equations (16) and (17), respectively*.

### 2.2. Dataset of *in silico* Pulse Waves

**Figure 2:**
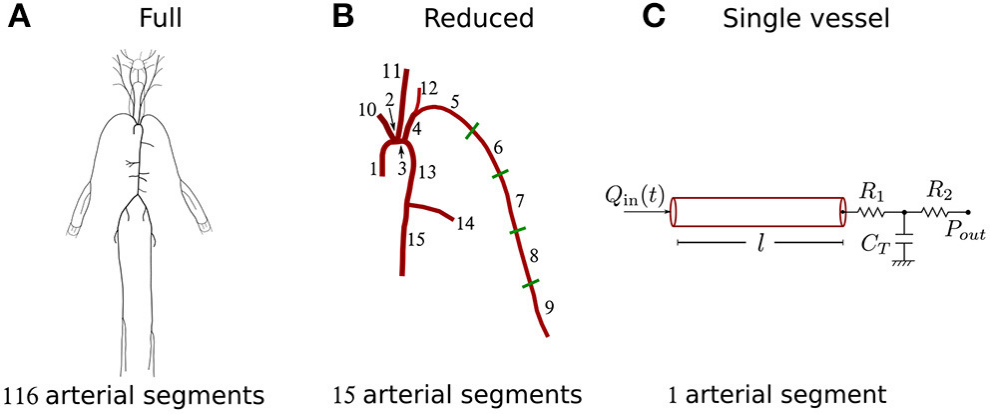
Schematic representation of the three models used in the study. **(A)** Full 116-artery model with cardiovascular properties taken from Charlton et al. (2019). **(B)** Reduced 15-artery model containing the aortic-brachial arterial path of the full model. **(C)** Single-vessel model of the brachial artery with the nomenclature used throughout the study. Tapering in the brachial artery of the full and reduced models was represented by five straight arterial segments of different radii. The single-vessel model allowed us to identify analytically the main physical determinants of ΔPP.

We used an existing dataset containing *in silico* cBP and pBP waves measured, respectively, at the aortic root and outlet of the brachial artery, and blood flow waves measured at the aortic root, in a group of 4,374 virtual subjects. Characteristics of the *in silico* group are given in Table 1. Pulse waves were simulated for subjects of each age decade, from 25 to 75 years old, using a 116-artery, one-dimensional (1-D) model of blood flow in the larger systemic arteries of the thorax, limbs, and head (Figure 2A). The model cardiovascular properties were identified through a comprehensive literature review and simulated pulse waves were verified by comparison against clinical data [see Charlton et al. (2019) for full details].

### 2.3. Blood Flow Models

Three blood flow models with decreasing level of mathematical complexity were employed in this study (Figure 2). Firstly, we used the 116-artery model of the systemic circulation described above. Blood pressure and flow waves at any point in the arterial network were simulated using our in-house, linear, one-dimensional (1-D) formulation, which enables analytical solutions in the frequency domain and has previously been verified against computational 1-D and 3-D solutions (Flores Gerónimo et al., 2016). This model—hereafter referred to as the “full model”—provided reference cBP and pBP waves to assess the accuracy of the other two simpler models.

**Figure 3:**
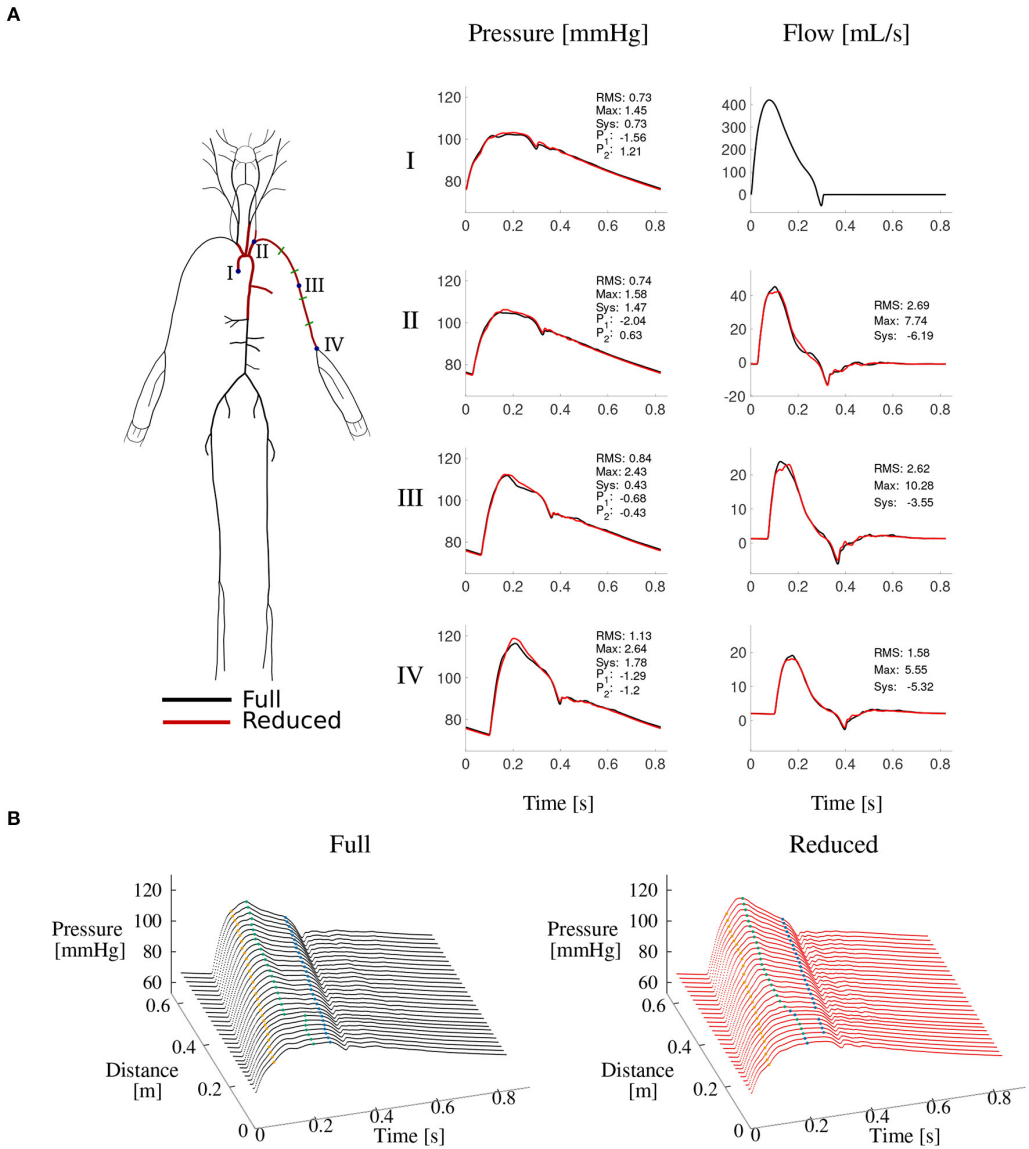
Reduced vs. full model pressure and flow waves. **(A)** Schematic representations of both models (left) and comparison of corresponding pressure and flow waves at four locations along the aortic-brachial arterial path (right). Each plot shows relative errors (expressed as percentages) for average (RMS), maximum (Max), systolic (Sys) and characteristic pressure points P_1_ and P_2_ calculated as described in Supplementary Section 1. **(B)** Pressure wave along the same arterial path, for the full (left) and reduced (right) model. Characteristic pressure points *P*_1_ (yellow), systolic pressure (green), and *P*_2_ (blue) are shown in each plot.

The second model simulated blood flow in the 15 arterial segments that make up the upper thoracic aorta and left brachial artery of the full model. This model hereafter referred to as the “reduced model”—has the same inflow waveform prescribed at the aortic root as the full model. Such inflow waveform was obtained by the *AorticFlowWave* script described in Charlton et al. (2019) for the desired inflow characteristics (heart rate, stroke volume and left ventricular ejection time). Figure 3A shows the inflow waveform for the 25 year-old baseline subject. The vascular network of the reduced model was obtained from the full model by lumping groups of arteries into optimized three-element windkessel (WK) models that preserved the total vascular compliance and resistance of the full model. Six sets of WK parameters were calculated, for the six terminal segments of the reduced model, from the flow and pressure waves produced by the full model using a gradient descent algorithm previously described in Fossan et al. (2018). Table 2 shows the vascular parameters of the reduced model that was able to reproduce pressure and flow waves along the aortic-brachial arterial path with relative errors smaller than 3 and 10%, respectively (Figure 3). All relative errors in this figure were calculated using the full model as a reference, as described in Supplementary Section 1.

**Table 2:** Vascular parameters of the reduced model.

**Arterial**	**Length**	***r*_d_**	***Eh***	***R*_1_**	***R*_2_**	***C*_Wk_**
**segment**	**(cm)**	**(mm)**	**(Pa m)**	**(mmHg s mL^−1^)**	**(mmHg s mL^−1^)**	**(mL mmHg^−1^)**
1. Asc. aorta	6.00	18.1	975.7	-	-	-
2. Aortic arch I	2.00	15.8	851.0	-	-	-
3. Aortic arch II	3.90	15.2	817.8	-	-	-
4. L. subclavian	3.40	6.4	346.4	-	-	-
5. Brachial I	8.44	5.4	292.3	-	-	-
6. Brachial II	8.44	4.8	262.9	-	-	-
7. Brachial III	8.44	4.3	234.4	-	-	-
8. Brachial IV	8.44	3.7	207.5	-	-	-
9. Brachial V	8.44	3.1	173.2	1.548	10.982	0.046
10. Brachiocephalic	3.40	8.6	461.1	0.123	4.523	0.252
11. L. Com. carotid	6.95	4.4	239.6	0.406	10.333	0.047
12. L. vertebral	3.70	2.2	152.2	1.582	57.410	0.008
13. Desc. Thor. aorta I	5.20	12.8	691.7	-	-	-
14. Intercostal	8.00	1.5	139.7	9.115	3.081	0.051
15. Desc. Thor. aorta II	10.40	9.7	520.3	0.125	0.964	0.683

*They were taken from the 25-year-old baseline subject in Charlton et al. (2019). Segment numbers are displayed in Figure 2B. The remaining parameters are: blood density ρ = 1, 060 Kg m^−3^, blood viscosity η = 2.5 × 10^−3^ Pa s and outflow pressure P_out_ = 33 mmHg. r_d_ is the diastolic luminal radius, E is the Young modulus, h is the wall thickness, R_1_, R_2_, and C_Wk_ are the two resistances and compliance, respectively, of the outflow windkessel models. L, left; Com, common; Desc, descending; Thor, thoracic*.

The reduced model provided accurate and workable analytical solutions for blood pressure and flow waves along the aortic-brachial arterial path. It was further simplified into a third model: a straight single-vessel model of the brachial artery which enabled us to identify analytically the main physical determinants of ΔPP, as described in the next sections.

#### 2.3.1. Single-Vessel Model

Blood pressure in the frequency domain, $\hat{p}\left(x,\omega \right)$, along the single-vessel model (Figure 2C) can be described by the summation of an attenuation, ${\hat{T}}_{1}\left(x,\omega \right)$, and an amplification, ${\hat{T}}_{2}\left(x,\omega \right)$, term based on the formulation described by Flores Gerónimo et al. (2016) (see Supplementary Section 2); i.e.,

(1) $$\begin{array}{l} \hat{p}={\hat{T}}_{1}+{\hat{T}}_{2}, \end{array}$$

where

(2) $$\begin{array}{l} \hat{T_{1}}=\left[\frac{\cos\left(k_{c}l\right)ẐM+\sin\left(k_{c}l\right)}{\cos\left(k_{c}l\right)-ẐM\sin\left(k_{c}l\right)}\right]\cos\left(k_{c}x\right)\frac{{\hat{Q}}_{\text{in}}}{M}, \end{array}$$

(3) $$\begin{array}{l} \hat{T_{2}}=-\sin\left(k_{c}x\right)\frac{{\hat{Q}}_{\text{in}}}{M}, \end{array}$$

with *x* the axial direction, ω the angular frequency, *l* the vessel length, ${\hat{Q}}_{\text{in}}$ the Fourier transform of *Q*_in_(*t*) the flow wave at the inlet, and

(4) $$\begin{array}{r} k_{c}^{2}\left(\omega \right)=\frac{i\omega C\eta }{A_{0}K\left(\omega \right)},\;M^{2}\left(\omega \right)=\frac{i\omega CA_{0}K\left(\omega \right)}{\eta },\\ Ẑ\left(\omega \right)=\frac{R_{1}+R_{2}-i\omega R_{1}R_{2}C_{\text{Wk}}}{1-i\omega R_{2}C_{\text{Wk}}}. \end{array}$$

η is the blood viscosity, *A*_0_ is the average luminal cross-sectional area, $C=\frac{3\pi r_{0}r_{\text{d}}^{2}}{2Eh}$ is the vessel compliance – with *E* the Young modulus, *h* the wall thickness, and *r*_0_ and *r*_d_ the average and diastolic luminal radii, respectively—*C*_Wk_ is the WK-model compliance, and *R*_1_ and *R*_2_ are the WK-model resistances. $K\left(\omega \right)=-\frac{\eta }{i\omega \rho }\left[1-\frac{2J_{1}\left(kr_{0}\right)}{kr_{0}J_{0}\left(kr_{0}\right)}\right]$ is the dynamic permeability where *J*_0_ and *J*_1_ are the Bessel functions of order zero and one, respectively, $k^{2}=\frac{i\omega \rho }{\eta }$ and ρ is the blood density. Inverse Fourier transforms can be applied to obtain time-domain blood pressure, *p*(*x, t*), and its attenuation and amplification components *T*_1_(*x, t*) and *T*_2_(*x, t*), respectively.

**Figure 4:**
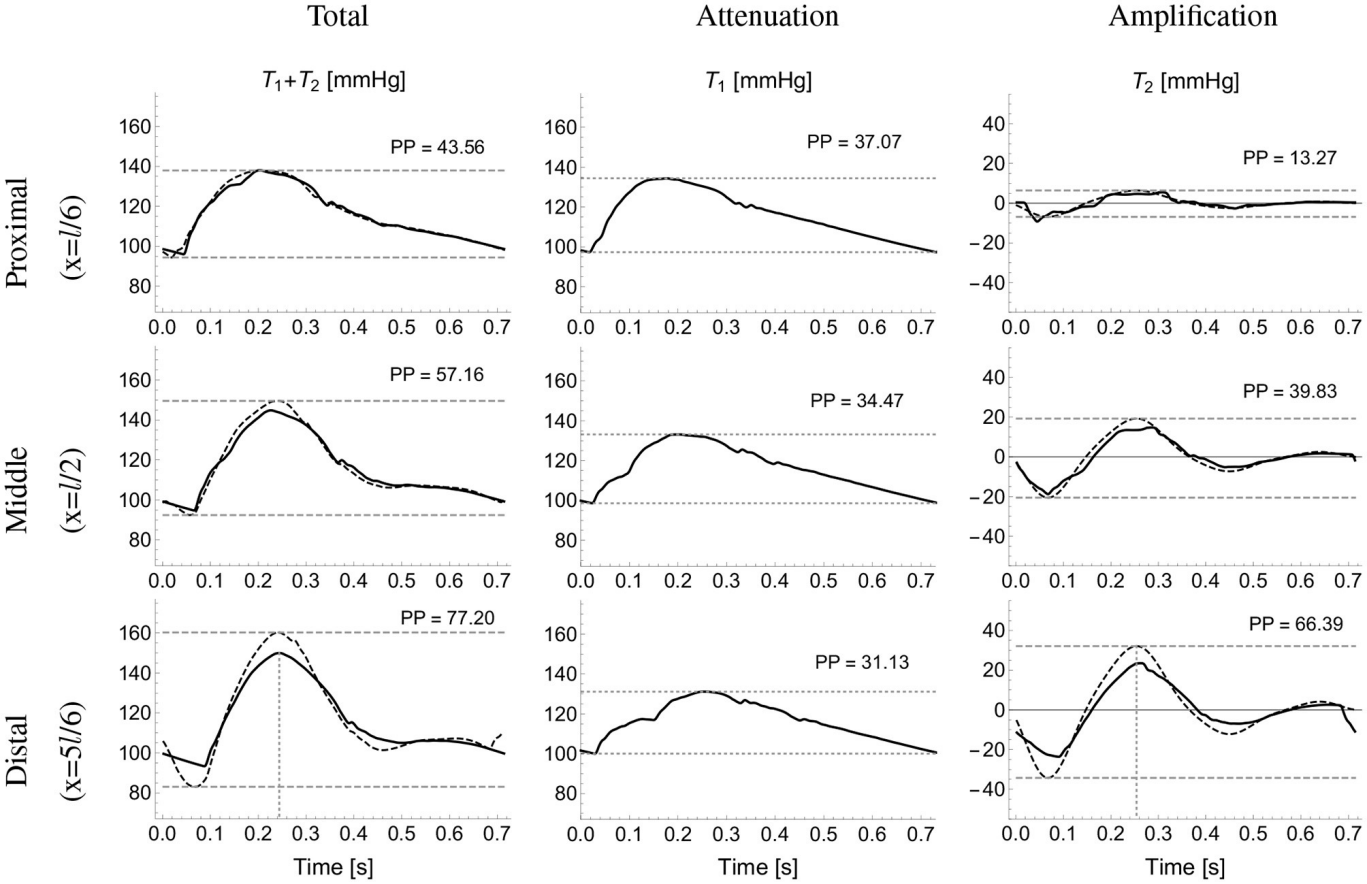
Analytical pressure wave decomposition. The total pressure wave calculated using the reduced model (first column) at proximal **(top)**, midpoint **(middle)**, and distal **(bottom)** sites along the brachial artery was decomposed into the attenuation, *T*_1_ (second column) and amplification, *T*_2_ (third column) terms calculated using the single-vessel model (Equations 2 and 3). The approximated total (sum of Equations 2 and 7) and *T*_2_ pressures (computed using Equation 7) are shown in dotted lines (first and third columns, respectively). Horizontal dashed lines highlight pulse pressure (PP) values. Vertical dashed lines (bottom plots) indicate the time of minimum d*Q*_in_/d*t*.

Figure 4 compares the pressure waves simulated by the reduced and single-vessel models along the brachial artery. It includes the terms *T*_1_ and *T*_2_ calculated using the single-vessel model (Equations 2 and 3), with *Q*_in_(*t*) the flow wave measured at the inlet of the brachial artery of the reduced model, *l* = 42.2 cm the total length of the brachial artery, *r*_0_ ≈ *r*_d_ = 4.3 mm, the average radii at diastolic pressure of the 5 arterial segments that make up the brachial artery in the reduced model, and *Eh* = 234 Pa m the average *Eh* value along the brachial artery, and *C* = 0.205 mm^2^/mmHg. The approximation for *r*_0_ ≈ *r*_d_ was used given that the difference between *r*_0_ and *r*_d_ was smaller than 2.5%, which allowed us to reduce the number of parameters in the model. The WK parameters *C*_Wk_, *R*_1_, *R*_2_ and *P*_out_ are equal to those of the WK model coupled to the outlet of the segment ‘Brachial V’ in the reduced model (see Table 2). We note that PP along the vessel is indeed slightly attenuated by *T*_1_ and considerably amplified by *T*_2_. Next, we derive an approximate expression for the amplification term *T*_2_.

#### 2.3.2. Approximate Amplification Term

A Taylor series expansion of the amplification term ${\hat{T}}_{2}\left(x,\omega \right)$ about ω = 0 yields

(5) $$\begin{array}{l} {\hat{T}}_{2}\approx -\frac{x\eta }{A_{0}K^{0}}\left[1-i\omega \left(\frac{\rho r_{0}^{2}}{6\eta }+\frac{C\eta x^{2}}{6A_{0}K^{0}}\right)\right]{\hat{Q}}_{\text{in}}, \end{array}$$

where $K^{0}=r_{0}^{2}/8$ is the steady state permeability. The time-domain solution of Equation (5) is given by

(6) $$\begin{array}{l} T_{2}\left(x,t\right)=-\frac{8x\eta }{\pi r_{0}^{4}}Q_{\text{in}}\left(t\right)-\frac{4x\eta }{3\pi r_{0}^{4}}\left(\frac{\rho r_{0}^{2}}{\eta }+\frac{8C\eta x^{2}}{\pi r_{0}^{4}}\right)\frac{dQ_{\text{in}}\left(t\right)}{dt}. \end{array}$$

For all segments of the aortic-brachial arterial path, the term containing *dQ*_in_/*dt* dominates. Moreover, the viscous time $\frac{\rho r_{0}^{2}}{\eta }$ is at least three orders of magnitude larger than $\frac{8C\eta x^{2}}{\pi r_{0}^{4}}$ (see Supplementary Section 2.1). As a result, Equation (6) can be further approximated as

(7) $$\begin{array}{l} T_{2}\left(x,t\right)=-\frac{4x\rho }{3\pi r_{0}^{2}}\frac{dQ_{\text{in}}\left(t\right)}{dt}. \end{array}$$

According to this equation the main determinants of *T*_2_ along an arterial segment are its radius, length, and the rate of change of *Q*_in_ with time. Moreover, the amplification term *T*_2_ is maximum when *dQ*_in_/*dt* is minimum and, hence, systolic blood pressure, P_Sys_(*x*), along an arterial segment can be estimated as

(8) $$\begin{array}{l} {\text{P}}_{\text{Sys}}\left(x\right)={\text{P}}_{\text{Sys}}^{\text{In}}-\frac{4x\rho }{3\pi r_{0}^{2}}{\frac{dQ_{\text{in}}}{dt}|}_{\text{min}}, \end{array}$$

where ${\text{P}}_{\text{Sys}}^{\text{In}}$ is the systolic pressure at the inlet and *dQ*_in_/*dt*|_min_ denotes the maximum temporal rate of decrease in late-systolic flow at the inlet. Assuming equal diastolic pressure at any point along the arterial segment, PP and ΔPP can be expressed as a function of the distance *x* from the inlet as

(9) $$\begin{array}{l} \text{PP}\left(x\right)=\text{P}{\text{P}}^{\text{In}}-\frac{4x\rho }{3\pi r_{0}^{2}}{\frac{dQ_{\text{in}}}{dt}|}_{\text{min}}, \end{array}$$

(10) $$\begin{array}{l} \Delta \text{PP}\left(x\right)=-\frac{4x\rho }{3\pi r_{0}^{2}}{\frac{dQ_{\text{in}}}{dt}|}_{\text{min}}, \end{array}$$

with PP^In^ the pulse pressure at the inlet. Figure 4 (third column) compares the exact *T*_2_ term along the brachial artery given by Equation (3) with the corresponding approximated term calculated using Equation (7). To avoid high-frequency noise, the time derivative of *Q*_in_ was filtered by computing the Fourier series of *dQ*_in_/*dt* and keeping terms up to 15 Hz to reproduce the filtered signal. The vertical dotted lines in the first and third columns indicate the time of the maximum temporal rate of decrease in late-systolic flow, *dQ*_in_/*dt*|_min_, which approximately corresponds to the time of peak pressure for the reduced model and the exact *T*_2_ term. The approximated pressure given by the sum of Equations (2) and (7), and shown on Figure 4 (first column) with dotted lines, overestimates systolic pressures calculated by the reduced model, shown on Figure 4 (first column) with continuous lines, by less than 6%.

### 2.4. Methodology for Estimating cPP

**Figure 5:**
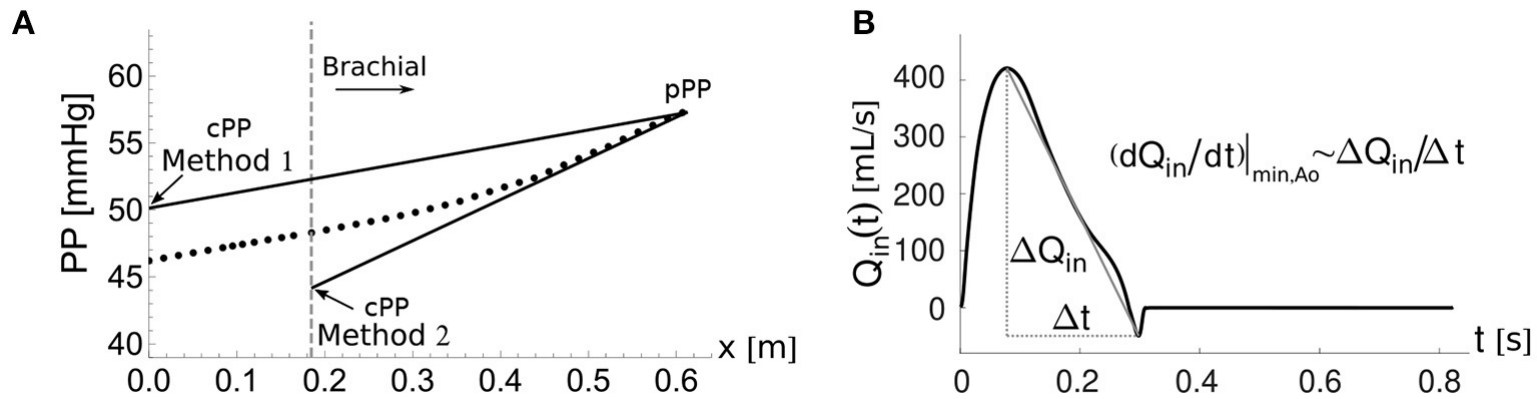
The two cPP estimation methods. **(A)** Pulse pressure, PP(x), along the aortic-brachial arterial path with distance, *x*, from the aortic root. The start of the brachial artery is indicated by the dashed vertical line. PP values calculated along the path using the reduced model (dots). Methods 1 and 2 estimate cPP (solid lines) based on the approximate expression for PP(x) given by Equation (11), starting from the PP value at the outlet of the brachial artery, pPP. **(B)** Approximate calculation of the maximum temporal rate of decrease in late-systolic flow, (*dQ*_in_/*dt*)|_min,Ao_, from the aortic flow waveform, *Q*_in_(*t*).

Based on the previous analytical expressions for the amplification term *T*_2_, two methods can be developed for estimating cPP at the aortic root from the flow at the aortic root or the brachial artery and pPP at the outlet of the brachial artery. Both are illustrated in Figure 5.

#### 2.4.1. Method 1

This method assumes that Equation (9) can be applied to the entire aortic-brachial arterial path as

(11) $$\begin{array}{l} \text{PP}\left(x\right)=\text{cPP}-\frac{4x\rho }{3\pi {\left(\bar{r}\right)}^{2}}{\frac{dQ_{\text{in}}}{dt}|}_{\text{min},\text{Ao}}, \end{array}$$

with *x* the distance from the aortic root, cPP the PP at the aortic root, (*dQ*_in_/*dt*)|_min,Ao_ the maximum temporal rate of decrease in late-systolic flow at the aortic root and $\bar{r}$ the mean radius of the aortic-subclavian arterial segments. Note that the suffix Ao stands for aortic. Equation (11) can be used to compute cPP from the PP at the brachial artery, pPP, as

(12) $$\begin{array}{l} \text{cPP}=\text{pPP}+\frac{4\;\rho l_{\text{Ao}-\text{Bra}}}{3\pi {\left(\bar{r}\right)}^{2}}{\frac{dQ_{\text{in}}}{dt}|}_{\text{min},\text{Ao}}, \end{array}$$

where *l*_Ao−Bra_ is the length of the aortic-brachial arterial path.

Equation (12) was used to estimate cPP for the *in silico* dataset. The following assumptions were used for the *in vivo* dataset. When using *in vivo* aortic flow data with low temporal resolution, such as the Doppler ultrasound data used in this study, (*dQ*_in_/*dt*)|_min,Ao_ can be approximated by (Figure 5B)

(13) $$\begin{array}{l} {\left(\frac{dQ_{\text{in}}}{dt}\right)|}_{\text{min},\text{Ao}}\approx {\left(\frac{\Delta Q_{\text{in}}}{\Delta t}\right)}_{\text{Ao}}, \end{array}$$

with Δ*Q*_in_ the end-systolic flow minus the peak flow and Δ*t* the time of end of systole minus the time of peak flow. *l*_Ao−Bra_ was estimated using the empirical expression (Sugawara et al., 2018)

(14) $$\begin{array}{r} l_{\text{Ao}-\text{Bra}}\left(\text{mm}\right)=37.9\times \text{sex}\left(\text{male}=1,\text{female}=0\right)\\ +1.4\times \text{age}\left(\text{years}\right)+2.5\times \text{height}\left(\text{cm}\right)-14.8. \end{array}$$

The radius of the aorta was used as a surrogate of the mean radius of the aortic-subclavian arterial segments, that is, $\bar{r}\approx r_{\text{Ao}}$.

#### 2.4.2. Method 2

This method assumes that all the change in PP along the aortic-brachial arterial path occurs in the brachial artery. As a result, Equation (12) becomes

(15) $$\begin{array}{l} \text{cPP}=\text{pPP}+\frac{4\;\rho \;l_{\text{Bra}}}{3\pi {\left(r_{\text{Bra}}\right)}^{2}}{\frac{dQ_{\text{in}}}{dt}|}_{\text{min},\text{Bra}}, \end{array}$$

where *l*_Bra_ and *r*_Bra_ are the length and mean radius of the brachial artery, and *dQ*_in_/*dt*|_min,Bra_ is the maximum rate of decrease of late systolic blood flow at the inlet of the brachial artery.

Equation (15) was used to estimate cPP for the *in silico* dataset. The following correlations and assumptions were used for the *in vivo* dataset. According to the *in silico* dataset, *r*_Bra_ is strongly correlated with the radius of the aortic root, *r*_Ao_ (both in cm) through

(16) $$\begin{array}{l} r_{\text{Bra}}=0.246\;r_{\text{Ao}}-0.04, \end{array}$$

with a correlation coefficient *R* = 0.98. Moreover, *dQ*_in_/*dt*|_min,Bra_ is correlated with the corresponding value at the aortic root (both in mL/s^2^), through

(17) $$\begin{array}{l} {\frac{dQ_{\text{in}}}{dt}|}_{\text{min},\text{Bra}}=0.0763{\frac{dQ_{\text{in}}}{dt}|}_{\text{min},\text{Ao}}+22, \end{array}$$

with *R* = 0.80. We therefore estimated the brachial radius and maximum temporal rate of decrease of brachial flow from the corresponding *in vivo* data at the aortic root.

The length of the brachial artery was estimated by multiplying the aortic-brachial length computed using Equation (14) by a factor of 0.73. This corresponds to the ratio of the brachial artery length to the total aortic-brachial length measured in the *in silico* dataset.

## 3. Results

### 3.1. Approximate Pressure Calculations Using the Single-Vessel Model

**Figure 6:**
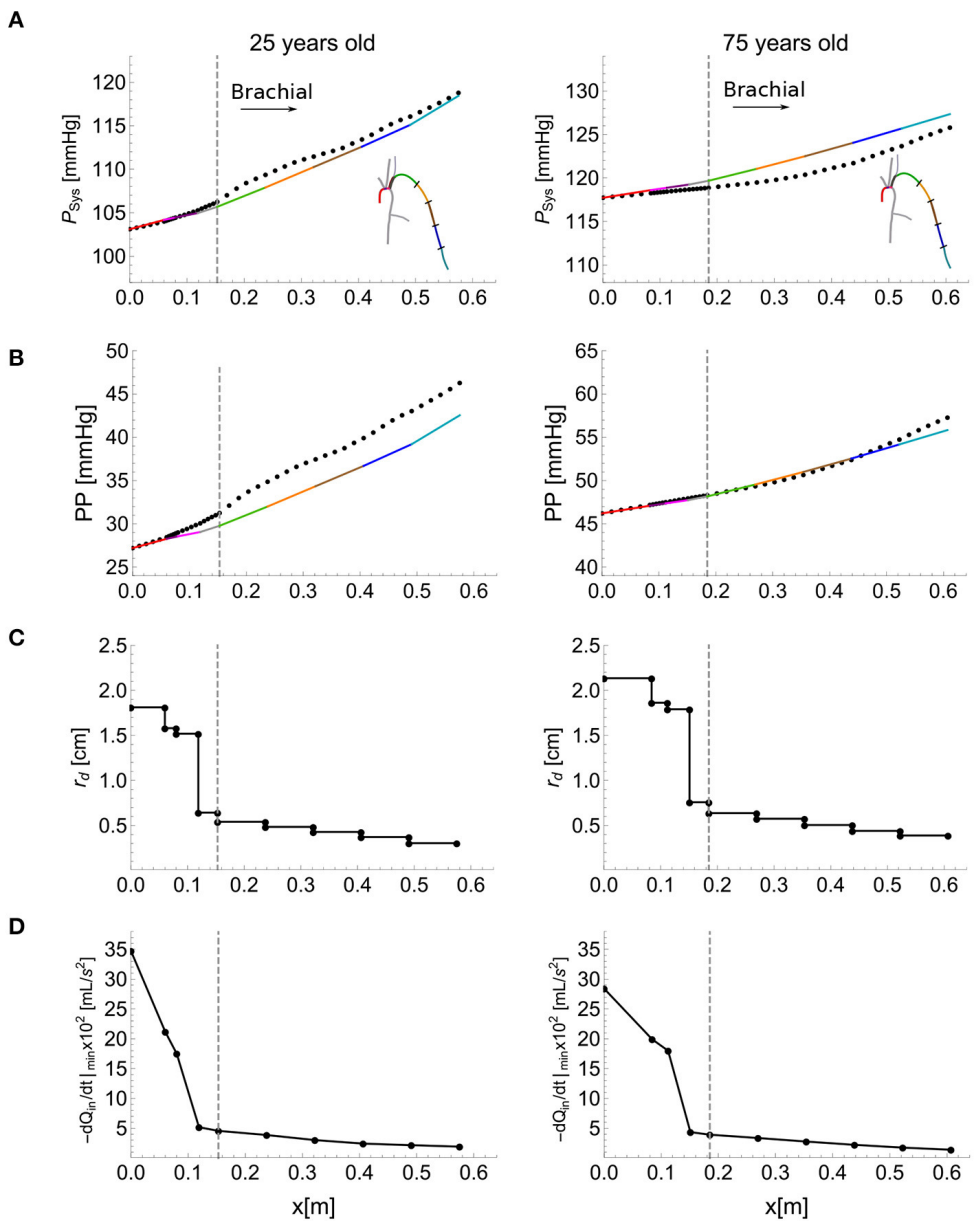
Calculation of systolic blood pressure, P_Sys_, and pulse pressure, PP, using the single-vessel model. P_Sys_ **(A)**, and PP **(B)** (solid lines) calculated using Equations (8) and (9), respectively. The dots show the corresponding values obtained by the reduced model. Luminal diastolic radius, *r*_d_ **(C)**, and maximum temporal rate of decrease in late-systolic flow, −d*Q*_in_/d*t*|_min_ from the reduced model **(D)** at the inlet and outlet of each segment of the aortic-brachial arterial path, for the baseline 25 (left) and 75 (right) year-old subject from the *in silico* dataset. Dashed vertical lines indicate the start of the brachial artery.

Figures 6A,B shows the ability of the single-vessel model to describe—through Equations (8) and (9), respectively—systolic pressure, P_Sys_, and PP for each segment along the aortic-brachial arterial path, for the 25 (left) and 75 (right) year-old baseline subjects of the *in silico* dataset. Results are compared with corresponding P_Sys_ and PP values obtained from reduced models of each subject. The inlet systolic pressure, ${\text{P}}_{\text{Sys}}^{\text{In}}$, in Equation (8) and inlet pulse pressure, PP^In^, in Equation (9) for the first arterial segment (ascending aorta) were taken from the values provided by the corresponding reduced model. For the remaining segments, ${\text{P}}_{\text{Sys}}^{\text{In}}$ and PP^In^ were assumed to be equal to the values calculated at the outlet of the adjacent upflow segment using Equations (8) and (9), with the luminal diastolic radius and maximum temporal rate of decrease in late-systolic blood flow shown in Figures 6C,D computed from the reduced model. Relative errors were smaller than 1.3% for P_Sys_ and 13.7% for PP for the 25 year-old *in silico* subject and 1.8% for P_Sys_ and 2.5% for PP for the 75 year-old *in silico* subject, along the aortic-brachial arterial path. The larger luminal radii and smaller rate of blood flow decrease in late systole in the 75-year-old subject led to a smaller pulse pressure amplification from aortic root to brachial artery, compared to the 25-year-old subject.

Starting from the PP values at the outlet of the brachial artery of the reduced model and assuming that the PP at the inlet of an arterial segment is equal to the PP at the outlet of the adjacent one, Equations (9) and (10) applied to each arterial segment from the brachial artery to the aortic root enabled us to calculate cPP and ΔPP with variations in cardiovascular properties. Cardiovascular properties variations for the 25-year-old *in silico* subjects led to relative errors smaller than 18% for cPP and 24% for ΔPP when the morphology of the aortic flow wave was modified to account for changes in stroke volume, heart rate and left ventricular ejection time (results are shown in the Supplementary Figure 3A). On the other hand, variations in total vascular resistance, total vascular compliance, total path length and average network radius (for the same aortic root waveform) led to relative errors smaller than 20% for cPP and 29% for ΔPP (Supplementary Figure 3B).

According to Equation (10), ΔPP along an arterial segment is proportional to the segment length and the maximum temporal rate of decrease in late systolic-flow; and inversely proportional to the square of the vessel radius. These proportionalities were verified in all the arterial segments of the aortic-brachial arterial path for the 25- and 75-years-old *in silico* subjects. Proportionalities for length and temporal rate of decrease in late systolic-flow exhibited linear correlations with the smallest correlation coefficient (R^2^) of 0.91. Proportionality in radius exhibited a power-decay with exponents of −1.73 and −2.18 for the 25- and 75-year-old *in silico* subjects, respectively, both exponents are close to the expected value of −2. Results are shown in the Supplementary Figure 2.

### 3.2. *In silico* Verification of cPP Estimation Methods

**Figure 7:**
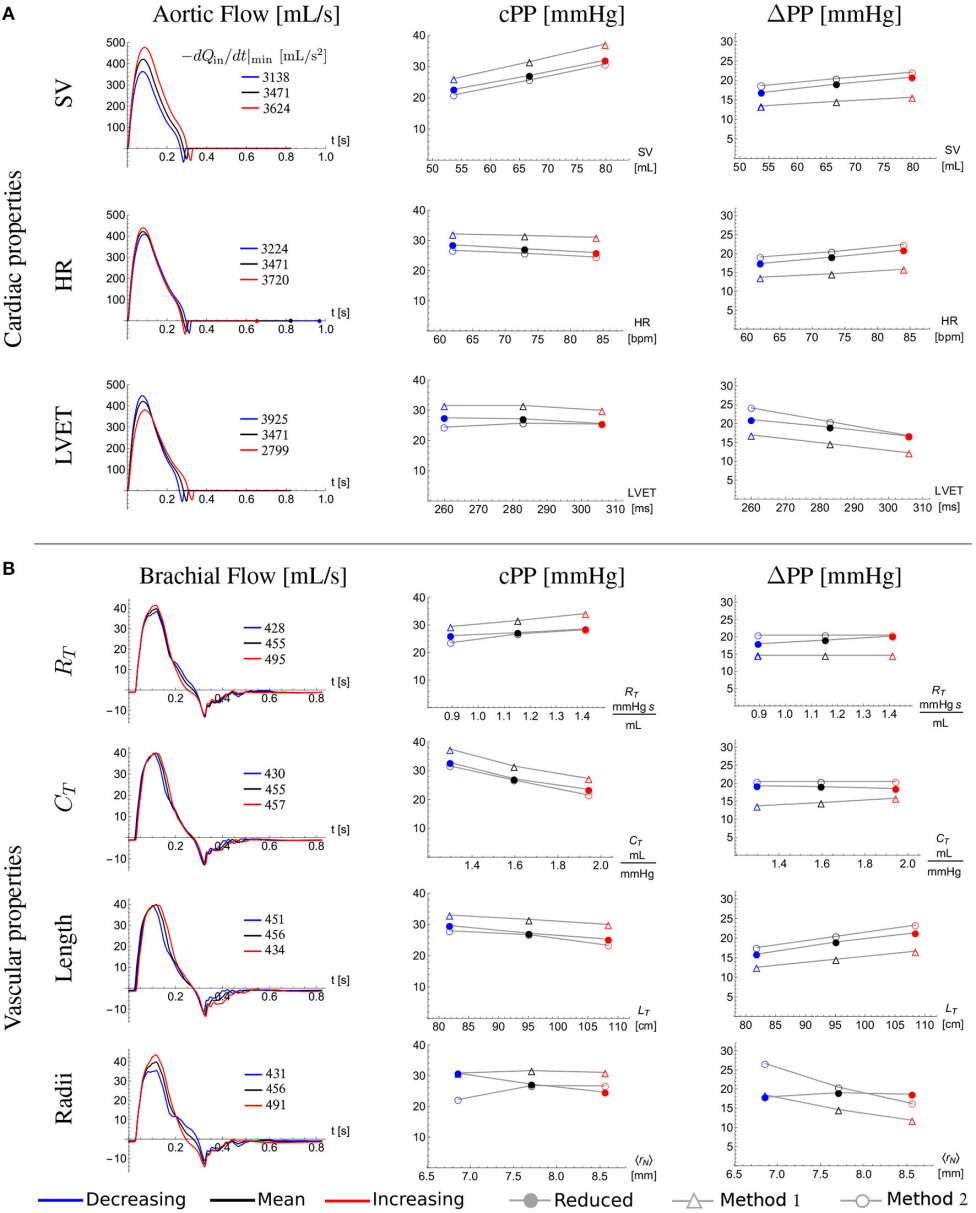
Effect of cardiovascular properties on central pulse pressure, cPP, and its amplification from the aortic root to the outlet of the brachial artery, ΔPP. Aortic root **(A)** and brachial artery **(B)** flow wave (first column), cPP (second column), and ΔPP (third column) for the 25-year-old baseline subject (black) and with a standard deviation (SD) decrease (blue) and a SD increase (red) in **(A)** stroke volume (SV), heart rate (HR) and left ventricular ejection time (LVET), and **(B)** total vascular resistance (*R*_*T*_) and total vascular compliance (*C*_*T*_); and with a 14% decrease (blue) and 14% increase (red) in the total network length (*L*_*T*_) and with a 11% decrease (blue) and 11% increase (red) in the average radius of the network (〈*r*_*N*_〉). The closed dots were calculated using the reduced model, the open triangles were calculated using Equation (12) of Method 1 and the open dots were calculated using Equation (15) of Method 2. Legends in the first column indicate the maximum temporal rate of decrease in late systolic-flow in mL/s^2^ for all flow waves.

Figure 7 shows the ability of the cPP estimation methods to describe—through Equation (12) for Method 1 and Equation (15) for Method 2—changes in cPP and ΔPP, respectively, with variations in cardiovascular properties. Mean and standard deviation (SD) for stroke volume, heart rate, left ventricular ejection time, total vascular resistance and total vascular compliance correspond to the 25-year-old *in silico* subjects. The length and the radius of each vessel of the 25-year-old baseline subject were changed by 14% and 11%, respectively. These percentages were calculated from the covariance (SD/Mean) of the aortic arch length (Bensalah et al., 2014) and brachial artery radius (van der Heijden-Spek et al., 2000), respectively. Both methods were able to capture changes in cPP and ΔPP produced by the variations in cardiovascular properties. Excluding radii variations, Method 1 led to relative errors smaller than 20% for cPP and 28% for ΔPP. Excluding radii variations, Method 2 led to smaller relative errors: 18% for cPP and 24% for ΔPP. Except for radii variation where a competing effect arises, approximate PP and ΔPP values followed the trends provided by the reduced model. For all the cardiac variations ΔPP increases with the maximum temporal rate of decrease in late-systolic flow and length; and is almost not affected by the total vascular resistance and total compliance as described by Equation (10) and consequently by Equations (12) and (15).

**Figure 8:**
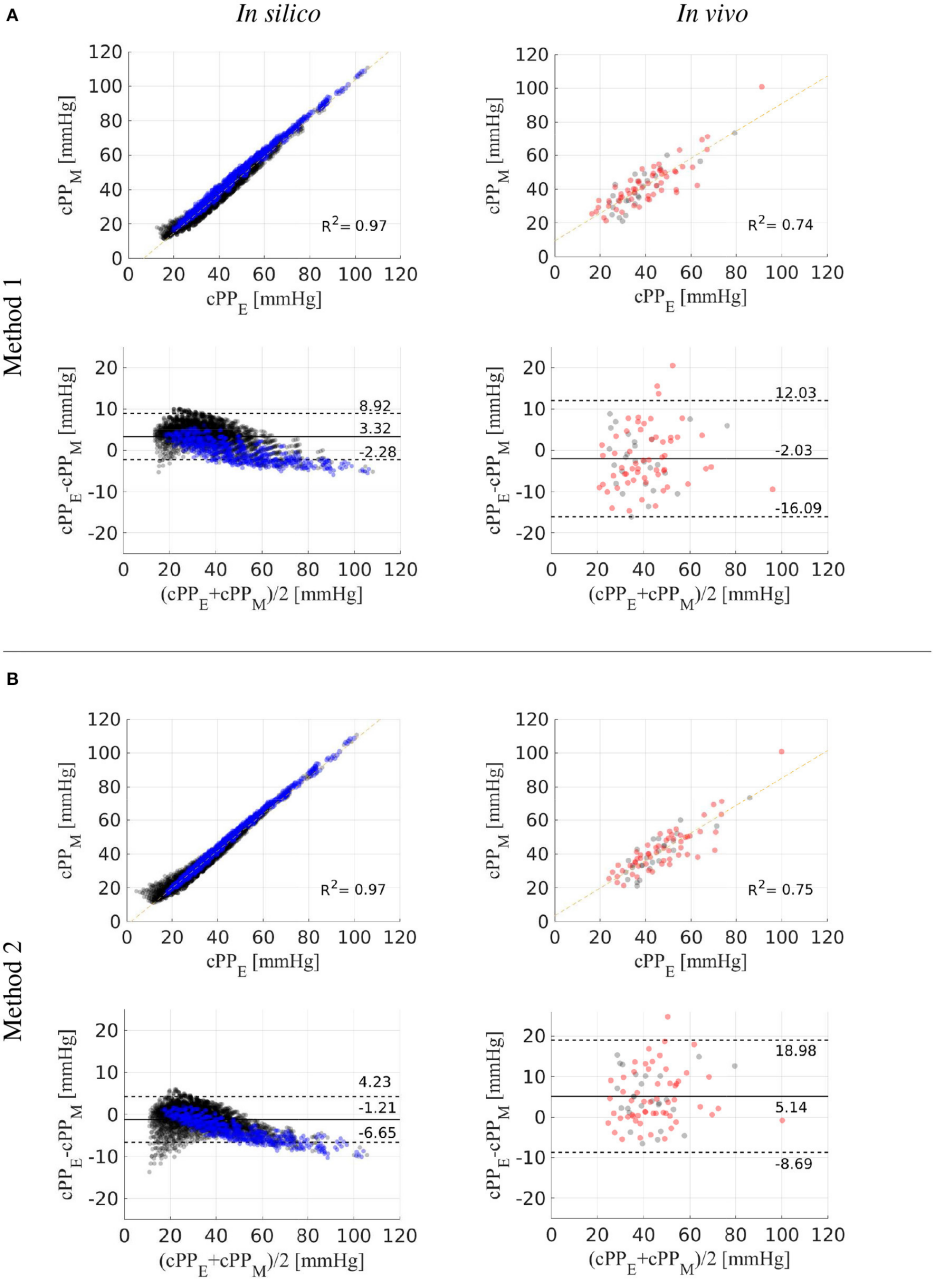
Estimated (cPP_E_) vs. measured (cPP_M_) central pulse pressure. Correlation and Bland-Altman plots comparing cPP_E_ values calculated using Methods 1 **(A)** and 2 **(B)** with (cPP_M_) values for the *in silico* (left) and *in vivo* (right) datasets. *In vitro* 75-year-old subjects are shown in blue dots. *In vivo* hypertensive subjects are shown in red dots. Dotted horizontal lines show the limits of agreement (±1.96*SD*) between estimated and reference cPP values.

We tested the accuracy of the two new cPP estimation methods on the dataset of *in silico* pulse waves (Figure 8, left). For the whole dataset, estimated cPP was strongly correlated with measured cPP, with R-squared (*R*^2^) values of 0.97 for both methods (Figures 8A,B, top). Overall, Method 1 (Equation 12) overestimated cPP (Figure 8A, bottom) and Method 2 underestimated cPP (Figure 8B, bottom). The mean ± SD error was 3.3 ± 2.8 mmHg for Method 1 and -1.2 ± 2.7 mmHg for Method 2. Estimated cPP values were closer to measured cPP values for Method 2. When only the 75-year-old subjects were considered (blue dots), the agreement between cPP obtained from Method 1 and measured values was much closer, with *R*^2^ values and mean ± SD errors of 0.99 and 0.1 ± 2.7 mmHg for Method 1, respectively, and 0.99 and −3.4 ± 2.7 mmHg for Method 2, respectively.

### 3.3. *In vivo* Verification of cPP Estimation Methods

We also tested the accuracy of the two new cPP estimation methods on the *in vivo* data (Figure 8, right). For the whole dataset, estimated cPP was strongly correlated with measured cPP, though with *R*^2^ values smaller than those obtained for the *in silico* dataset: 0.74 for Method 1 and 0.75 for Method 2. Method 1 led to a smaller mean error than the one obtained for the *in silico* dataset (−2.0 vs. 3.3 mmHg). However, the SD was greater in the *in vivo* dataset (7.0 vs. 2.8 mmHg). For Method 2, both the mean error and SD were larger in the *in vivo* dataset (5.1 ± 6.9 vs. −1.2 ± 2.7 mmHg). For both methods, no considerable differences in mean, SD or *R*^2^ values were observed between normotensive (Method 1: −2.4 ± 6.7 mmHg, R^2^ = 0.72; Method 2: 4.9 ± 6.4 mmHg, R^2^ = 0.75) and hypertensive (Method 1: −1.9 ± 7.4 mmHg, R^2^ = 0.74; Method 2: 5.3 ± 7.4 mmHg, R^2^ = 0.74) subjects.

## 4. Discussion

We have developed and tested two methods for estimating cPP from aortic or brachial blood flow and peripheral blood pressure. Both methods originated from a model of blood flow in the larger arteries of the systemic circulation based on physical principles (conservation of mass and linear momentum). The mathematical complexity of this full model was simplified based on the physics of blood flow, resulting in a reduced model of blood flow in the upper thoracic aorta and left brachial artery that allows for workable analytical solutions in the frequency domain. This reduced model enabled us to obtain an approximation for blood pressure, in time domain, along each segment of the aortic-brachial arterial path from which the main cardiovascular determinants of ΔPP could be identified, leading to the two new cPP estimation methods. Both methods were able to (i) capture changes in cPP and ΔPP produced by variations in cardiovascular properties and (ii) estimate cPP with mean differences smaller than 3.3 ± 2.8 mmHg on *in silico* data for different age groups (25–75 years old) and 5.1 ± 6.9 mmHg on *in vivo* data for normotensive and hypertensive subjects.

### 4.1. Main Cardiovascular Determinants of Pulse Pressure Amplification

We have obtained a simple expression highlighting the main cardiovascular determinants of ΔPP along an arterial segment. According to Equation (10), ΔPP is directly proportional to the distance along the arterial segment and the maximum temporal rate of decrease in late-systolic flow, and inversely proportional to the square of the vessel radius. Vascular geometry, therefore, plays a very important role in changing PP from central to peripheral arterial sites. This result is in agreement with previous studies in which ΔPP was associated with higher height and, hence, greater vessel length (Asmar et al., 1997; Camacho et al., 2004) and greater tapering and, hence, greater decrease in vessel radius (Belardinelli and Cavalcanti, 1992; Mynard and Smolich, 2015). Mynard and Smolich (2015) found, using computational modeling, that brachial artery tapering is a major factor leading to wave intensity amplification in the arm. They suggested that this may explain why amplification of wave intensity, which is directly related to the rate of change of pressure with time (and hence PP), was not found in other regions where the degree of tapering was lower. Our analytical results further support this finding. We have shown that changes in PP mainly occur in the brachial artery rather than in the aorta (Figure 6B), despite the rate of decrease in systolic flow being greater in the aorta than in the brachial artery (Figure 6D). This is because (i) the brachial artery is longer than the upper thoracic aorta and (ii) the vessel radius, which has an inversely quadratic contribution to ΔPP in Equation (10), is much smaller in the brachial artery (Figure 6C).

Equation (10) highlights the predominant role that flow wave morphology in late systole plays in ΔPP. Cardiac properties such as stroke volume (SV), heart rate (HR) and left ventricular ejection time (LVET) introduce variations in late-systolic aortic flow resulting in changes in ΔPP (Figure 7). In particular, increasing SV or HR and decreasing LVET augments the rate of decrease in late-systolic aortic flow and causes a greater ΔPP, as predicted by Equation (10) and consequently by Equations (12) and (15). This result is in agreement with previous studies underlining the potential importance of ventricular dynamics in determining ΔPP (O'Rourke, 1970; Asmar et al., 1997; Gaddum et al., 2017); e.g., O'Rourke (1970) found that, in human subjects, ΔPP increases with decreasing LVET. Equation (10) provides a mechanistic explanation of this result since a smaller LVET would lead to a greater temporal rate of decrease in late-systolic flow and, hence, greater ΔPP.

Interestingly, ΔPP is independent of the total compliance of the network (Figure 7B), again in agreement with Equation (10) and despite cPP decreasing with the increasing compliance. Indeed, cPP is mainly determined by total arterial compliance and left ventricular ejection dynamics affecting SV (Vennin et al., 2017). Except for radii variation, where the effects of the radius and the maximum temporal rate of decrease in late systolic-flow (induced by radii variation) are competing, approximate PP and ΔPP values followed the trends provided by the reduced model (Figure 7).

Our analytical results are in concordance with the experimental observation of a linear increase in ΔPP with the distance along an arterial segment (Gaddum et al., 2017) and further analysis of cardiac variables could be performed in experimental setups to corroborate our analytical results. Observed tendencies of *in vivo* measurements, like the difference in ΔPP with gender (Segers et al., 2009), can be analyzed in terms of our results, namely, the brachial artery length correlates with the height which in turn is significantly different with gender (Max Roser and Ritchie, 2019), however, *in vivo* measurements of radius and proximal flow at the brachial artery would be needed to corroborate the correlations between ΔPP and its determinants.

### 4.2. Aging and Pulse Pressure Amplification

Aging is associated with increased PP (Pinto, 2007) and decreased ΔPP (Herbert et al., 2014). It has been shown to increase PP more rapidly in central rather than distal arteries and, consequently, it has been suggested that ΔPP attenuation is predominantly caused by an increase in central systolic BP (cSBP) which, in turn, is induced by a rise in arterial stiffness (Safar et al., 2002). Other studies have highlighted the importance of ventricular dynamics—in addition to arterial stiffness—in determining PP (Fok et al., 2014b; Vennin et al., 2017; Li et al., 2019). Our study shows that the decrease in ΔPP with age is mainly caused by the increase in arterial radius and decrease in the rate of change of aortic flow with time in late systole (see Equation 10) strongly correlated with left ventricular ejection dynamics. The mechanisms behind the age-related increase in diameter are still unclear, though they may be the result of a compensatory adaptation to plaque formation and/or increases in wall thickness (Thijssen et al., 2015).

### 4.3. Central Pulse Pressure Estimation Methods

We have developed two methods that estimate cPP from measurements of the aortic flow wave and pPP that can be obtained by non-invasive means; e.g., Doppler ultrasound or magnetic resonance imaging for aortic flow and an oscillometric device for pPP. We have tested both methods on a dataset of *in silico* pulse waves and on a cohort of normotensive and hypertensive subjects, which covered a wide range of pressure wave morphologies and cPP values including those seen in hypertensive subjects (Table 1). Stronger linear correlations between estimated and reference cPP values were observed for the in *in silico* data compared to the *in vivo* data, the same was true for the smaller values for the mean and SD of the errors for cPP estimates (Figure 8). This is due to the larger experimental errors of the *in vivo* measurements, compared to the uncertainties of the *in silico* data due to model assumptions.

Recent (2017) clinical guidelines for the validation of non-invasive central blood pressure devices propose a mean absolute difference ≤ 5 mmHg with a SD ≤ 8 mmHg compared with the reference cSBP (Sharman et al., 2017). Method 1 led to mean absolute differences within recommended values (2.0±7.0 mmHg), though Method 2 did not (5.1±6.9 mmHg). Direct implementation of Method 2 requires measurements of the flow wave, vessel length and vessel radius in the brachial artery, which were not available for our *in vivo* cohorts and could lead to smaller mean absolute differences within recommended values. Indeed, Method 2 led to smaller differences than Method 1 when tested using *in silico* measurements in the brachial artery.

By using transfer functions, the influence of the large arteries and peripheral load on pulse pressure amplification has been studied (Karamanoglu et al., 1995). Using a single-tube transmission line model enables personalization of some of the transfer function parameters, improving the accuracy of the estimated cBP waveform (Swamy et al., 2009; Hahn et al., 2012; Gao et al., 2016; Natarajan et al., 2017). Further improvement in cBP assessment could be achieved by including additional patient-specific information to transfer functions based on the results of our study; i.e., the geometry of the brachial artery and the flow morphology. It is worth mentioning that our model approach differs from the lossless transmission line model approach used by Swamy et al. (2009), Hahn et al. (2012), Gao et al. (2016), Natarajan et al. (2017). First, starting from continuum mechanics (fluid dynamics, and a model for vessel elasticity), our approach accounts for the spatial and temporal variations of blood pressure and flow in the brachial artery. Second, it accounts for viscous losses along the vessel. Third, it decomposes pressure along the vessel into space-varying attenuation and amplification terms, whereas the transmission line model does a decomposition into forward and backward waves that are independent of position (the distance between the peripheral and central positions is introduced by adding a time delay). The main determinants of ΔPP are also different in both models: the pulse transit time and reflection coefficient in the transmission line model, and the inflow wave morphology and arterial geometry in our model. Notably, the main determinants of ΔPP in our model are independent of peripheral vascular properties.

Machine learning approaches could also be used to estimate cPP from aortic or brachial flow and pPP. However, like transfer function methods, they should be trained on datasets covering the large range of physiological and pathophysiological conditions encountered in healthy and diseased subjects, to make them widely applicable. The ability to numerically generate datasets containing hemodynamic data for thousands of virtual subjects (Charlton et al., 2019) could facilitate this process.

### 4.4. Limitations

This study is subject to several important limitations. Our non-invasive measurements of pressure and the flow were not simultaneous and subject to experimental error. Carotid pressure is also an imperfect surrogate of aortic pressure and subject to calibration errors. Both Methods 1 and 2 require a flow wave measurement in addition to the peripheral pulse pressure. Calculation of the maximum temporal rate of flow decrease in late systole requires differentiation of the flow wave, which can be challenging when working with noisy flow waves; e.g., those acquired by Doppler ultrasound. To facilitate this calculation, we have approximated this maximum temporal rate by the slope given from peak flow to end of systolic flow (see Figure 5B). Further validation of both methods using more accurate techniques for measuring flow, such as magnetic resonance, would be valuable, as well as further validation of Method 2 using measurements of blood flow and vascular geometry in the brachial artery. We tested both methods only in normotensive subjects and in otherwise hypertensive subjects. Although comparison with the dataset of *in silico* pulse waves has provided a wider range of pressure and flow wave morphologies and hemodynamic conditions, indicating that both methods would be equally valid in pathological conditions, such as systolic hypertension in older subjects and in heart failure, this needs testing prospectively.

### 4.5. Perspectives

Assessment of cPP has been shown to be of greater predictive value for cardiovascular outcomes than brachial PP. It is, therefore, essential to understand the hemodynamic determinants of PP amplification. The results that we have presented here show that blood flow is a key determinant of PP amplification, confirming the importance of measuring flow and the key role played by ventricular ejection. Therefore, conditions and drugs that influence cardiac function may influence pulse wave morphology independent of arterial function. Moreover, the two methods proposed here allow for a more regular assessment of a patient's cPP, due to their non-invasive nature which removes the risk of complications due to cardiac catheterization.

## 5. Conclusion

We have identified the main determinants of pulse pressure amplification—highlighting the important role of flow morphology—and presented two methods based on the physics of blood flow for estimating central pulse pressure from non-invasive measurements of aortic flow and peripheral blood pressure. We have tested both methods on *in silico* data for different age groups (25–75 years old) and *in vivo* data for normotensive and hypertensive subjects. Our approach could improve cardiovascular function assessment in clinical cohorts for which aortic ultrasound or magnetic resonance imaging data are available.

## Data Availability Statement

The raw data supporting the conclusions of this article will be made available by the authors, without undue reservation.

## Author Contributions

All authors contributed to the article and approved the submitted version.

## Conflict of Interest

The authors declare that the research was conducted in the absence of any commercial or financial relationships that could be construed as a potential conflict of interest.
