# Supplementary material for: Estimating Central Pulse Pressure From Blood Flow by Identifying the Main Physical Determinants of Pulse Pressure Amplification
Source: Front Physiol. 2021 Feb 23;12:608098. doi: 10.3389/fphys.2021.608098 (PMC7940670; doi:10.3389/fphys.2021.608098)
Supplement: Supplementary file 1 [file Presentation_1.pdf]

Supplementary Material

1 ERROR CALCULATIONS

The following relative error metrics for blood pressure, P, and flow, Q, waves were used to assess the accuracy of the reduced model,

$$\mathcal{E}_P^{\mathrm{RMS}} = \sqrt{\frac{1}{n}\sum_{i=1}^{n}\left(\frac{P_i^{\mathrm{RM}} - P_i^{\mathrm{FM}}}{P_i^{\mathrm{FM}}}\right)^2}, \qquad \mathcal{E}_Q^{\mathrm{RMS}} = \sqrt{\frac{1}{n}\sum_{i=1}^{n}\left(\frac{Q_i^{\mathrm{RM}} - Q_i^{\mathrm{FM}}}{\max_j(Q_j^{\mathrm{FM}})}\right)^2}, \tag{S1}$$

$$\mathcal{E}_P^{\mathrm{Max}} = \max_i \frac{P_i^{\mathrm{RM}} - P_i^{\mathrm{FM}}}{P_i^{\mathrm{FM}}}, \qquad \mathcal{E}_Q^{\mathrm{Max}} = \max_i \frac{Q_i^{\mathrm{RM}} - Q_i^{\mathrm{FM}}}{\max_j(Q_j^{\mathrm{FM}})}, \tag{S2}$$

$$\mathcal{E}_P^{\mathrm{Sys}} = \frac{\max(P^{\mathrm{RM}}) - \max(P^{\mathrm{FM}})}{\max(P^{\mathrm{FM}})}, \qquad \mathcal{E}_Q^{\mathrm{Sys}} = \frac{\max(Q^{\mathrm{RM}}) - \max(Q^{\mathrm{FM}})}{\max(Q^{\mathrm{FM}})}, \tag{S3}$$

where P_i^{RM} and Q_i^{RM} are the values obtained with the reduced model at a given spatial location and time point i ($i = 1, \ldots, n$), and $n = 823$ is the number of temporal points over the cardiac cycle. At the same spatial location and time point i, P_i^{FM} and Q_i^{FM} are the pressure and flow calculated using the full model. $\mathcal{E}_P^{\mathrm{RMS}}$ and $\mathcal{E}_Q^{\mathrm{RMS}}$ are the root mean square relative errors for pressure and flow waves; $\mathcal{E}_P^{\mathrm{Max}}$ and $\mathcal{E}_Q^{\mathrm{Max}}$ are the maximum relative errors for pressure and the flow; and $\mathcal{E}_p^{\mathrm{Sys}}$ and $\mathcal{E}_Q^{\mathrm{Sys}}$ are the errors in systolic pressure and flow, respectively. Flow errors were normalised by the maximum flow over the cardiac cycle to avoid division by small values. The following relative error metrics were used for the pressure characteristic points P_1 and P_2 computed using the *PulseAnalyse* script described in Charlton et al. (2019),

$$\mathcal{E}_P^{P_1} = \frac{P_1^{\mathrm{RM}} - P_1^{\mathrm{FM}}}{P_1^{\mathrm{FM}}} \quad \text{and} \quad \mathcal{E}_P^{P_2} = \frac{P_2^{\mathrm{RM}} - P_2^{\mathrm{FM}}}{P_2^{\mathrm{FM}}}. \tag{S4}$$

2 SINGLE-VESSEL MODEL

Blood pressure in the frequency domain, $\hat{p}(x,\omega)$ along a single vessel coupled to a windkessel (WK) model is given by (Flores Gerónimo et al. (2016))

$$\hat{p} = \left[\frac{\sin(k_c l)\cos(k_c x)}{\cos(k_c l)} - \sin(k_c x)\right]\frac{\hat{Q}_{\mathrm{in}}}{M} + \left[\frac{\cos(k_c x)}{\cos(k_c l)(\cos(k_c l) - \hat{Z}M\sin(k_c l))}\right]\hat{Q}_{\mathrm{in}}\hat{Z}, \tag{S5}$$

with the parameters described in Section 2.3.1 *Single-Vessel Model*. Equation S5 can be decomposed into an attenuation ($\hat{T}_1(x,\omega)$) and amplification ($\hat{T}_2(x,\omega)$) term, namely

$$\hat{T}_1 = \left[\frac{\sin(k_c l)\cos(k_c x)}{\cos(k_c l)}\right]\frac{\hat{Q}_{\mathrm{in}}}{M} + \left[\frac{\cos(k_c x)}{\cos(k_c l)(\cos(k_c l) - \hat{Z}M\sin(k_c l))}\right]\hat{Q}_{\mathrm{in}}\hat{Z} \tag{S6}$$

$$= \left[\frac{\cos(k_c l)\hat{Z}M + \sin(k_c l)}{\cos(k_c l) - \hat{Z}M\sin(k_c l)}\right]\cos(k_c x)\frac{\hat{Q}_{\mathrm{in}}}{M}, \tag{S7}$$

$$\hat{T}_2 = -\sin(k_c x)\frac{\hat{Q}_{\text{in}}}{M}. \tag{S8}$$

2.1 Amplification term

The time-domain solution of a low frequency approximation for the amplification term $T_2(x,t)$ is given by

$$T_2(x,t) = -\frac{8x\,\eta}{\pi r_0^4}Q_{\text{in}}(t) - \frac{4x\,\eta}{3\pi r_0^4}\left(\frac{\rho r_0^2}{\eta} + \frac{8C\eta x^2}{\pi r_0^4}\right)\frac{dQ_{\text{in}}(t)}{dt}. \tag{S9}$$

Figure S1 shows each term in Equation (S9) at the outlet of the ascending aorta and brachial artery. The

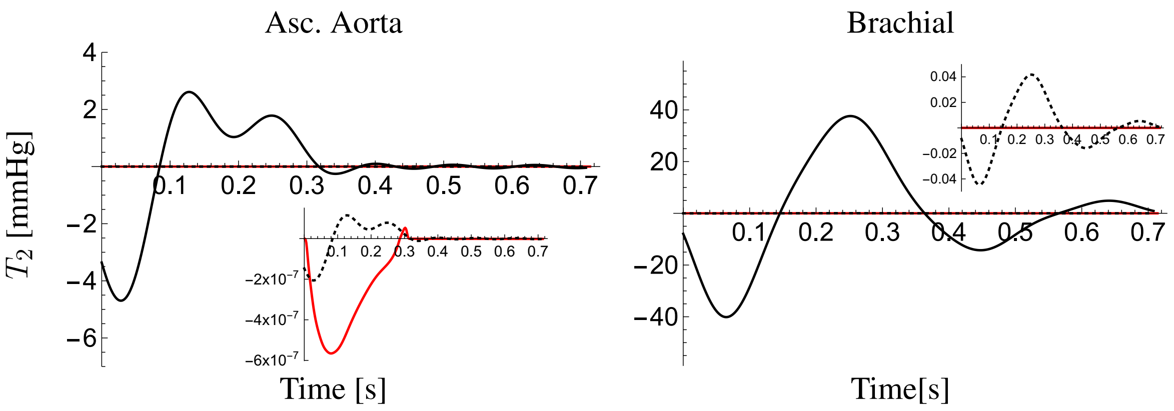

Figure S1. Components of the amplification term, $T_2(x,t)$, with time at the outlet of the ascending aorta (left) and brachial artery (right) of the 25-year-old subject in the *in silico* dataset. The $\rho r_0^2/\eta$ term in Equation (S9) is shown in the outer plots. The inflow (red lines) and $8C\eta x^2/\pi r_0^4$ (dotted lines) terms are shown in the inner plots.

continuous black line corresponds to the term with the characteristic time $\frac{\rho r_0^2}{\eta}$, which clearly dominates over the other two. As a result, T_2 can be simplified to

$$T_2 = -\frac{4x\,\rho}{3\pi r_0^2}\frac{dQ_{\text{in}}(t)}{dt}. \tag{S10}$$

The same behaviour is observed in all the segments along the aortic-brachial arterial path. To reduce the number of parameters in the models and given that the differences between the average (r_0) and diastolic (r_{d}) luminal radii are small, we have used $r_0 \approx r_{\text{d}}$ in all our calculations.

3 AMPLIFICATION TERM VERIFICATION

According to Equation (10) in Section 2.3.2 *Approximate Amplification Term*, pulse pressure amplification (ΔPP), along an arterial segment is proportional to the segment length (l), and the maximum temporal rate of decrease in late systolic-flow ($-dQ_{\text{in}}/dt|_{min}$); and inversely proportional to the square of the luminal diastolic radius (r_{d}). To verify these proportionalities, for all 729 *in silico* subjects aged 25 years old and all 729 aged 75 years old, we extracted ΔPP, r_{d}, l, $-dQ_{\text{in}}/dt|_{min}$, for all the arterial segments in the aortic-brachial arterial path. Then to analyze the effect of changing one variable at a time, we used $-\Delta\text{PP}\; r_{\text{d}}^2/(dQ_{\text{in}}/dt|_{\text{min}})$ as the dependent variable to observe the effect of the length (Figure S2A),

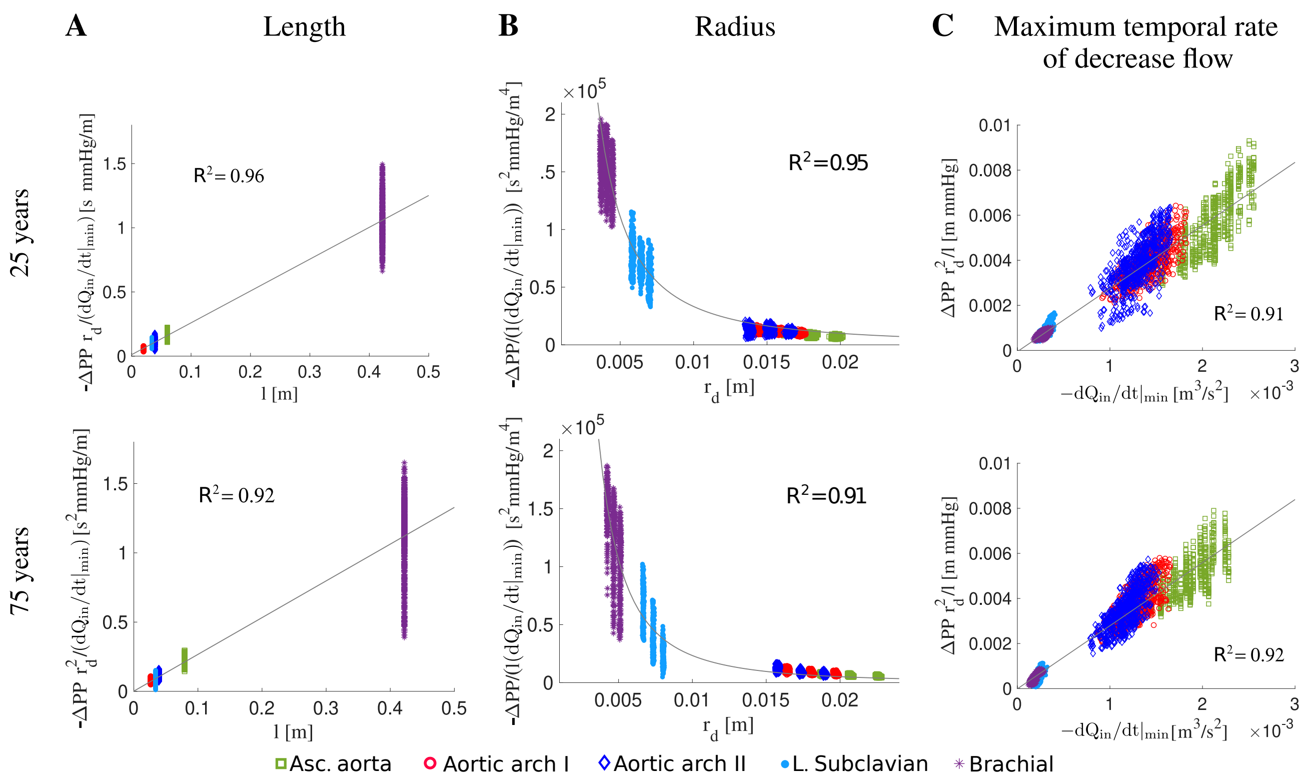

Figure S2. Determinants of pulse pressure amplification, ΔPP. Each marker corresponds to an arterial segment within the aortic-brachial arterial path. Results are shown for all the 25- (top) and 75- (bottom) year-old *in silico* subjects. Following Equation (10) in Section 2.3.2 *Approximate Amplification Term*, $-\Delta\text{PP}\ r_\text{d}^2/(dQ_\text{in}/dt|_\text{min})$ is used to observe the effect of the length, l, (first column), $-\Delta\text{PP}/(l(dQ_\text{in}/dt|_\text{min}))$ is used to observe the effect of the luminal diastolic radius, r_d, (second column) and $\Delta\text{PP}\ r_\text{d}^2/l$ is used to observe the effect of the maximum temporal rate of decrease in late systolic-flow, $-dQ_\text{in}/dt|_\text{min}$, (third column). Grey lines indicate the best linear fits for length and maximum temporal rate of decrease in late systolic-flow; and the best power-law fit for the diastolic radius. For each plot, the correlation coefficient (R^2) is shown.

$-\Delta\text{PP}/(l(dQ_\text{in}/dt|_\text{min}))$ to observe the effect of the luminal diastolic radius (Figure S2B) and $\Delta\text{PP}\ r_\text{d}^2/l$ to observe the effect of the maximum temporal rate of decrease in late systolic-flow (Figure S2C).

Length and temporal rate of decrease in late systolic-flow exhibited linear correlations, shown with grey lines in Figures S2A and S2C, respectively. The smallest correlation coefficient was $\text{R}^2 = 0.91$. Radius exhibited a power-law decay with exponents of -1.73 and -2.18 for the 25- and 75- year-old *in silico* subjects, respectively (shown with a grey line in Figure S2B). Both exponents are close to the expected value of -2.

4 *IN SILICO* VERIFICATION OF CPP ESTIMATION WITH THE SINGLE VESSEL MODEL

This section contains the results for cPP and ΔPP estimation with variations in cardiovascular properties using the single-vessel model. Mean and standard deviation (SD) for stroke volume, heart rate, left ventricular ejection time, total vascular resistance and total vascular compliance correspond to the 25 year-old *in silico* subjects. The length and the radius of each vessel of the 25-year-old baseline subject were changed by 14% and 11%, respectively. These percentages were calculated from the

covariance (SD/Mean) for the aortic arch length (Bensalah et al. (2014)) and brachial artery radius (van der Heijden-Spek et al. (2000)), respectively. Results are shown in Figure S3.

REFERENCES

Bensalah, M. Z., Bollache, E., Kachenoura, N., Giron, A., De Cesare, A., Macron, L., et al. (2014). Geometry is a major determinant of flow reversal in proximal aorta. *A J Physiol-Heart Circ Physiol* 306, H1408–H1416. doi:10.1152/ajpheart.00647.2013

Charlton, P., Mariscal Harana, J., Vennin, S., Li, Y., Chowienczyk, P., and Alastruey, J. (2019). Modelling arterial pulse waves in healthy ageing: a database for in silico evaluation of haemodynamics and pulse wave indices. *Am J Physiol Heart Circ Physiol* 317, H1062–H1085. doi:10.1152/ajpheart.00218.2019

Flores Gerónimo, J., Alastruey, J., and Corvera Poiré, E. (2016). A novel analytical approach to pulsatile blood flow in the arterial network. *Ann Biomed Eng* 44, 3047–3047. doi:10.1007/s10439-016-1625-3

van der Heijden-Spek, J. J., Staessen, J. A., Fagard, R. H., Hoeks, A. P., Boudier, H. A. S., and Bortel, L. M. V. (2000). Effect of age on brachial artery wall properties differs from the aorta and is gender dependent. *Hypertension* 35, 637–642. doi:10.1161/01.HYP.35.2.637

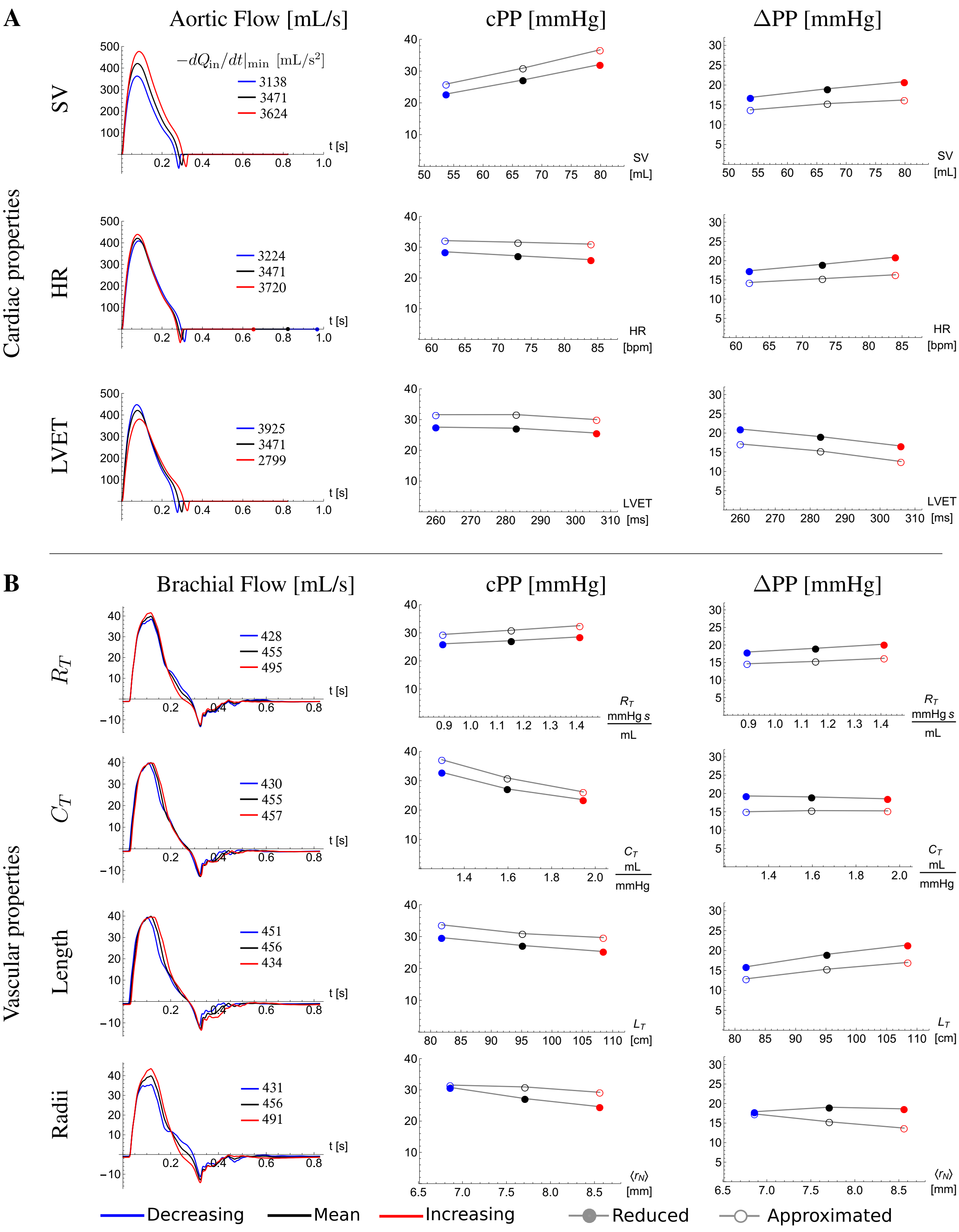

Figure S3. Effect of cardiovascular properties on central pulse pressure, cPP, and its amplification from the aortic root to the outlet of the brachial artery, ΔPP. Aortic root **(A)** and brachial artery **(B)** flow wave (first column), cPP (second column), and ΔPP (third column) for the 25-year-old baseline subject (black) and with a standard deviation (SD) decrease (blue) and a SD deviation increase (red) in **(A)** stroke volume (SV), heart rate (HR) and left ventricular ejection time (LVET), and **(B)** total vascular resistance (R_T) and total vascular compliance (C_T); and with a 14% decrease (blue) and 14% increase (red) in the total network length (L_T) and with a 11% decrease (blue) and 11% increase (red) in the average radius of the network ($\langle r_N \rangle$). The closed dots were calculated using the reduced model and the open dots were calculated using Equations (9) and (10) of the single-vessel model. Legends in the first column indicate the maximum temporal rate of decrease in late systolic-flow in mL/s^2 for all flow waves.
